# Adenovirus type 36 regulates adipose stem cell differentiation and glucolipid metabolism through the PI3K/Akt/FoxO1/PPARγ signaling pathway

**DOI:** 10.1186/s12944-019-1004-9

**Published:** 2019-03-21

**Authors:** Yi Jiao, Xiaodi Liang, Jianfei Hou, Yiliyasi Aisa, Han Wu, Zhilu Zhang, Nuerbiye Nuermaimaiti, Yang Zhao, Sheng Jiang, Yaqun Guan

**Affiliations:** 1 Department of Biochemistry, Preclinical Medicine College, Xinjiang Medical University, No. 393 Xinyi Road, Urumqi, 830011 Xinjiang China; 2 Department of Endocrinology, the First Affiliated Hospital of Xinjiang Medical University, No. 393 Xinyi Road, Urumqi, 830011 Xinjiang China; 3 Department of Burn and Plastic Surgery, the First Affiliated Hospital of Xinjiang Medical University, Urumqi, 830011 Xinjiang China

**Keywords:** Adenovirus type 36, Adipose stem cell, Cell differentiation, Glucose and lipid metabolism, PI3K/Akt/FoxO1/PPARγ signaling pathway

## Abstract

**Background:**

This study aims to investigate the molecular mechanism of Adenovirus type 36 (Ad36) in adipocyte differentiation and glucolipid metabolism.

**Methods:**

Rat obesity model was established by Ad36 infection and high-fat diet, respectively. Comparison of the body weight, clinical biochemical indicators, insulin sensitivity and lipid heterotopic deposition between these two models was performed. Ad36-induced adipocyte in vitro model was also established. The binding rate of FoxO1, PPARγ and its target gene promoter was detected using ChIP. The mRNA and protein expression levels of PPARγ and downstream target genes were detected by RT-PCR and Western blot, respectively. Oil red O staining was used to measure differentiation into adipocyte. Wortmannin (WM), inhibitor of PI3K, was used to act on Ad36-induced hADSCs.

**Results:**

Ad36-induced obese rats did not exhibit disorders in blood glucose and blood TG, insulin resistance and lipid ectopic deposition. The expression of Adipoq, Lpin1 and Glut4 in the adipose tissue increased. Oil red O staining showed that Ad36 induced the differentiation of hAMSCs into human adipocytes in vitro. During this process, the binding rate of FoxO1 and PPARγ promoter regions was weakened. However, the binding rate of the transcription factor PPARγ to its target genes Acc, Adipoq, Lpin1 and Glut4 was enhanced, and thus increased the protein expression of P-FoxO1, PPARγ2, ACC, LPIN1, GLUT4 and ADIPOQ. The PI3K inhibitor Wortmannin reduced the expression of P-Akt, P-FoxO1 and PPARγ2, thereby inhibiting adipogenesis of hADSC.

**Conclusion:**

Ad36 may promote fatty acid and triglyceride synthesis, and improve insulin sensitivity by affecting the PI3K/Akt/FoxO1/PPARγ signaling pathway.

## Introduction

Although obesity is a high risk factor for type 2 diabetes mellitus (T2DM) and cardiovascular disease, not all obese individuals are accompanied by insulin resistance or increased risk of T2DM and cardiovascular disease [1]. Recent studies [2–4] have shown that viral infections are related to obesity. Eight kinds of viruses have been experimentally found to be associated with obesity [5]. Among them, SMAM-1 and adenovirus type 36 (Ad36) infections can cause human obesity [6]. Ad36-infection induces an increase in body weight and body fat, but causes metabolic phenotype changes like lower blood lipids, easily-controlled blood glucose [1, 7]. Ad36 infection decreases leptin expression and increases the expression of the anti-inflammatory cytokine adiponectin [8, 9], which play important roles in glucose and lipid metabolism. However, the mechanism underlying the effect of Ad36 induced adipocyte differentiation and changes in glucose and lipid metabolism is unclear.

Ad36 E4orf1 is the protein encoded by the first open reading frame of early gene E4 and plays an important role in the development of obesity [10]. The C-terminal region of E4orf1 has a PDZ domain binding motif (PBM), which binds to Drosophila Disc Large-1 (Dlg-1) to form a complex [11]. The complex will activate the PI3K/Akt signaling pathway [10, 12]. The activity of the transcription factor FoxO1 is regulated by Akt [13, 14]. FoxO1 it can bind to the PPARγ promoter region to inhibit the expression of PPARγ gene [15]. We speculate that Ad36 may induce adipocyte differentiation and changes of glucose and lipid metabolism by regulating PI3K/Akt/FoxO1/PPARγ signaling pathway.

In this study, obese rat models were induced by Ad36 and high fat diet (HFD), respectively. The differences in body fat distribution, clinical biochemical indicators, insulin sensitivity and gene expression were compared. The regulation of glucose and lipid metabolism by Ad36 was further explored in vitro. In this study, the effects of transcription factors FoxO1 and PPARγ on their target genes were detected by chromatin immunoprecipitation (ChIP). The level of these genes was further detected by qRT-PCR and Western blot. Wortmannin, an inhibitor of PI3K, was used to further investigate the role of transcription factors FoxO1 and PPARγ and their downstream target genes in promoting adipocyte differentiation and lipid accumulation, and in regulating glucose and lipid metabolism.

## Materials and methods

### Animals and grouping

A total of 80 SPF grade 4-week-old Wistar rats (weighing 180–220 g) were provided by the Experimental Animal Center of Xinjiang Medical University. After a week of adaptation, they were randomly divided into 4 groups: Control group, Ad2 infection group (the negative control group), HFD group and Ad36 infection group (*n* = 20 for each group). Rats in the Ad2 infection group and the Ad36 infection group were intraperitoneally injected with 5 × 10^6^ PFU virus suspension, respectively. The control group and the HFD group were intraperitoneally injected with an equal volume of physiological saline. The rats in the control, Ad36 and Ad2 groups were fed with normal diet, and the rats of the HFD group were fed with high-fat-diet. The weight of the rats was measured weekly. Serum neutralization test was used to determine whether Ad36 and Ad2 were successfully infected. All animal experiments were conducted according to the ethical guidelines of the Institutional Animal Care and Use Committee of the First Affiliated Hospital of Xinjiang Medical University.

### Glucose and insulin tolerance test

For intraperitoneal glucose tolerance test (IPGTT), overnight fasted (12 h) rats received a glucose load (4 m g/kg body weight) intraperitoneally. For insulin tolerance test (ITT), rat fasted for 4 h were injected intraperitoneally with 1 U/kg insulin (Actrapid, Novo Nordisk). For both tests, blood glucose was measured at the tail vein with a glucose oxidase assay (BioVision, Mountain View, CA, USA).

### Sample collection and HE staining

After 20 weeks of treatment, the rats were fasted, anesthetized with 2.5% sodium pentobarbital, and sacrificed. Blood was collected from abdominal aorta. Serum was isolated after centrifugation at 3000 rpm for 10 min. Adipose tissue was collected in liquid nitrogen immediately and then transfer to − 80 °C freezer or fixed in 10% formalin solution. The liver was also collected and fixed in 10% formalin solution. As follows, first the tissue was dehydrated with a 70, 80, 90, 95 and 100% ethanol gradient and cleared with xylene. Then, the tissue was embedded in paraffin and cut into 3–4 μm sections. The tissue sections were stained with HE reagent. The cross-sectional area of intact adipocytes was measured using Motic Image Advanced 3.0 software (Motic medical diagnostic systems Co., Ltd., Xiamen, China), and finally the average value was calculated.

### Blood biochemical analysis

Blood glucose was measured by glucose oxidase method. Serum triglyceride was determined by glycerol phosphate oxidase-peroxidase method. Serum insulin was detected by radioimmunoassay, and serum adiponectin level was detected by ELISA. The insulin resistance index (HOMA-IR) was determined using the minimal steady-state model. The formula was: HOMA-IR = fasting blood glucose (mmol/L) × fasting insulin (mIU/L)/22.5.

### Isolation and adipogenic induction of hADSC

The human adipose derived stem cells (hADSCs) were isolated from the subcutaneous adipose tissues of patients who underwent plastic surgery in the department of burn and plastic surgery, the First Affiliated Hospital of Xinjiang Medical University. Clinical and biochemical examinations confirmed that these subjects did not have acute inflammation, cancers, endocrine diseases or infectious diseases. The study was conducted under the hospital ethics committee approval and informed consent from individual was obtained. The methods of hADSCs isolation and identification were performed according to a previous protocol [16]. For the adenovirus infection, hADSCs were first starved for 6 h, and then infected with 5 MOI Ad36 or Ad2 in a 37 °C, 5% CO_2_ incubator for 1 h. Ad2 was a negative control group. The virus suspension was discarded, and the cells were cultured with low-glucose DMEM containing 10% FBS. The medium was discarded after the first 48 h, and then changed every other day until further analysis.

### Chromatin immunoprecipitation (ChIP)

The hADSCs were infected with or without Ad2 or Ad36 for 48 h and 72 h, respectively. Magna ChIP G Chromatin Immunoprecipitation Kit (Millipore Corporation, Billerica, MA, USA) was used to identify the regions of the genome associated with FoxO1 or PPARγ. The CHIP assays were performed as previously described, with modifications [17] Briefly, cells were incubated for 10 min in 1% formaldehyde and then quenched with glycin for 5 min. After washing and cellular and nuclear lysis, chromatin breakdown was performed with an Ultrasonic Cell Disruptor (LANYI Shanghai, China). A preclear step was performed (1 h at 4 °C with 40 μl of magnetic beads) before overnight incubation of chromatin and beads with anti-FoxO1 or anti- PPARγ antibodies (CST, Danvers MA, USA). Cells were also submitted to a no-antibody condition (mock condition) to exclude non-specific binding. DNA purification was performed using the silica columns provided with the kit (Magna ChIP G, Millipore). The purified DNA samples were used as templates for qPCR detection. The primers used were shown in Table 1.

**Table 1:** The primers of CHip-PCR

Gene	Primer sequence (5′ → 3′)
Human Pparγ-forward	CCAGGAATAGACACCGAAAGA
Human Pparγ-reverse	TGAGGGGCGTGAACGTACT
Human Acc-forward	CGAACGCAGCAATCAAATAA
Human Acc-reverse	CGTCCAGCCTTGACATCTG
Human Glut4-forward	AAGACCAGTGAGGGTGATGG
Human Glut4-reverse	CGCTCTCCTCGTTTATCCAG
Human Adipoq-forward	GTGCTCCCTTCTGAAGCACT
Human Adipoq-reverse	GAAGATGGGGCAAAAGTCAA
Human Lpin1-forward	TTCACGCGAGACAGTGGTAG
Human Lpin1-reverse	CTGTCTCCAGAGGAGGTTGG

The following PCR conditions were used: denaturation at 94 °C for 10 min, followed by 35 cycles of 94 °C for 20 s and 60 °C for 1 min. Each sample was repeated 3 times. The bands were quantified by relative quantitative analysis and calibrated with the amount of input.

### Intervention of Wortmannin

The experiment was performed with the following groups: blank control group, DMSO control group, Ad2 infection group, Ad2+ WM group, Ad36 infection group and Ad36 + WM group. The hADSCs were infected with 5 MOI of Ad2 and Ad36, respectively. Ad2 + WM group and Ad36 + WM group were intervened with 200 nmol/L WM simultaneously with adenovirus infection. The complete media were changed every two days. On the 6th day, oil red O staining or mRNA and protein expression detection was performed. Each group had three duplicate wells and three times of experiment was repeated.

### Oil red O staining

The hADSCs were treated as described above, and the complete culture medium in the petri dish was discarded. The cells were washed 3 times with PBS, fixed with 4% paraformaldehyde for 30 min. After washing with deionized water, oil red O solution was added and incubated for 15 min. Then, the cells were observed with an inverted microscope and photographed.

### Determination of glucose concentration in the culture supernatant and intracellular triglyceride content

Cells were cultured with phenol red-free medium. After 0, 2, 4, 6, and 8 days of Ad36 or Ad2 infection, cell supernatant was collected, and the medium glucose concentration was determined with the glucose oxidase assay (BioVision, Mountain View, CA, USA). The cells were lysed with RIPA lysis, and the intracellular triglyceride content was measured with the triglyceride assay kit (BioSino, Beijing, China). The content of glucose and triglyceride were corrected by the total protein content in the culture dish.

### Real-time quantitative PCR (qPCR)

RNA was extracted from adipose tissues and adipocytes using TRIZOL reagent (Invitrogen, USA). The reverse transcription was performed with Reverse Transcription System (Promega, Madison, WI, USA). Real-time PCR was performed on an ABI 7500 Real-time PCR System (Applied Biosystems, Foster City, CA, USA). The qPCR was performed with the SYBR Select Master Mix (ABI, Carlsbad, CA, USA), and all reactions were performed in triplicate. The following PCR condition was used: denaturation at 95 °C for 10 min, followed by 40 cycles of 95 °C for 15 s and 60 °C for 1 min. The primers for qPCR were synthesized by Sangon Biotech (Shanghai, China) and the primer sequences were listed in Table 2.

**Table 2:** The primers of qRT-PCR

Gene	Primer sequence (5′ → 3′)
Human actin-Forward	GAGCACAGAGCCTCGCCTTT
Human actin-Reverse	GAGCGCGGCGATATCATCA
Human FoxO1-forward	GGCTGAGGGTTAGTGAGCAG
Human FoxO1-reverse	AAAGGGAGTTGGTGAAAGACA
Human Pparγ-forward	GGGATCAGCTCCGTGGATCT
Human Pparγ-reverse	TGCACTTTGGATCTCTTGAAGTT
Human Adipoq-forward	AACATGCCCATTCGCTTTACC
Human Adipoq-reverse	TAGGCAAAGTAGTACAGCCCA
Human Lpin1-forward	GCTGGAGAGGGAGAGAGGAT
Human Lpin1-reverse	GGTGGCTGCTGTTAGGACA
Human Acc-forward	TCCGCACTGACTGTAACCAC
Human Acc-reverse	GCGACTTCCATACCGCATTA
Human Glut4-forward	TGGGCGGCATGATTTCCTC
Human Glut4-reverse	GCCAGGACATTGTTGACCAG
Rattus actin-Forward	AGCCATGTACGTAGCCATCC
Rattus actin-Reverse	ACCCTCATAGATGGGCACAG
Rattus Acc-forward	TACAACGCAGGCATCAGAAG
Rattus Acc-reverse	TGTGCTGCAGGAAGATTGAC
Rattus APN-forward	AATCCTGCCCAGTCATGAAG
Rattus APN-reverse	TCTCCAGGAGTGCCATCTCT
Rattus Lpin1-forward	CCATTCACAGCGAGTCTTCA
Rattus Lpin1-reverse	TGGAAGGGGAATCTGACTTG
Rattus Glut4-forward	GCTTCTGTTGCCCTTCTGTC
Rattus Glut4-reverse	TGGACGCTCTCTTTCCAACT

### Western blot analysis and immunoprecipitation

Cells were lysed with the RIPA, and the total protein concentration was determined with the BCA method. Protein samples were separated by SDS-PAGE, and then transferred onto a PVDF membrane (Bio-Rad, Hercules, CA, USA). After blocking with 5% blocking buffer at room temperature for 2 h, the membrane was incubated with the primary antibody (1:1000 dilution; CST, Danvers, MA,USA) at 4 °C overnight. The membrane was then incubated with horseradish peroxidase (HRP)-conjugated secondary antibody (ZSGB-BIO, Beijing, China) at room temperature for 1 h. Color development was performed with the electrochemiluminescence (ECL) method. The protein bands were scanned by the image acquisition system (Bio-Rad), and the images were analyzed with the Image J software.

### Statistical analysis

The statistical analysis was performed with the statistical software SPSS 17.0 (SPSS Inc., Chicago, IL, USA). Analysis of variance (ANOVA) or the Student’s *t* test was used. Data were expressed as mean ± SEM (standard error of mean). A *P* value less than 0.05 was considered as statistically significant.

## Results

### Ad36 infection increases rat body weight but does not affect insulin sensitivity

**Fig. 1:**
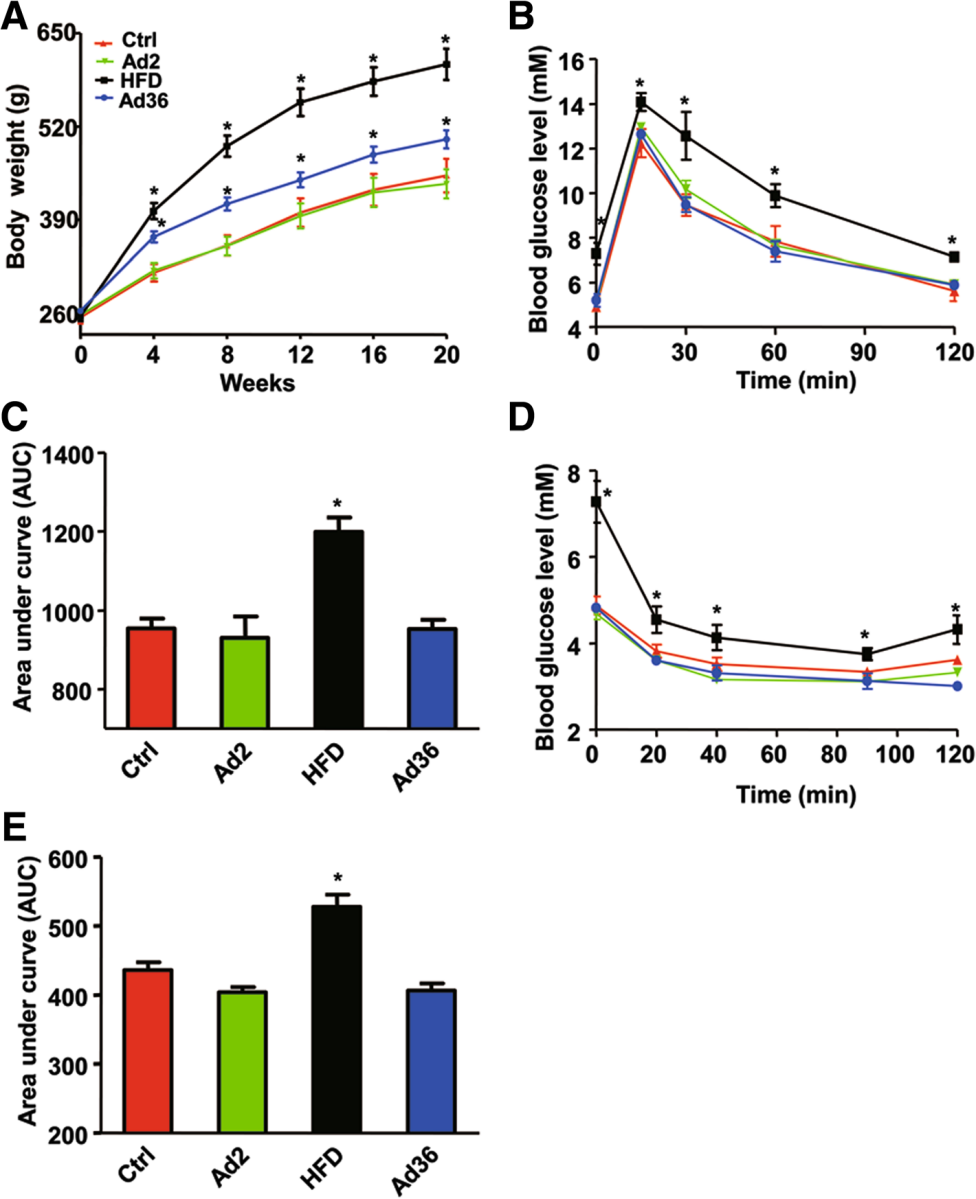
The effect of Ad36 infection on the body weight and insulin sensitivity of rats. The rats were intraperitoneally injected with 5 × 10^6^ CFU virus suspension, and the (**a**) body weight was measured; (**b**) the blood glucose levels and (**c**) the area under curve (AUC) of IPGTT; and (**d**) the blood glucose levels and (**e**) the area under curve (AUC) of ITT were shown. HFD was used as the positive control, and Ad2 was used as the negative control. * *P* < 0.05, compared with the control group; ^#^ *P* < 0.05, compared with the HFD group

To determine the effect of Ad36 on insulin sensitivity, clinical parameters were measured, and the IPGTT and ITT were performed. At Week 0, the rats in each group had similar body weights. Compared with the blank control group, the body weights of the HFD group and the Ad36 group were significantly increased at Week 4, 8, 12, 16 and 20 (*P* < 0.05, Fig. 1a). Blood biochemical results showed that compared with the control group, the blood glucose, triglyceride, serum insulin, and HOMA-IR were significantly increased in the HFD -induced obese rats, and serum adiponectin levels were decreased (Table 3) (*P* < 0.05). Ad36-induced obese rats had reduced triglyceride concentrations, while serum adiponectin levels were elevated (*P* < 0.05). However, blood glucose, insulin, and HOMA-IR levels were not statistically different from the Control group (Table 3).

**Table 3:** The comparison of biochemical parameters

Groups	Glucose (mM)	Triglyceride (mM)	Insulin (mU/L)	Adiponectin (μg/mL)	HOMA-IR
Blank control	5.90 ± 1.38	0.84 ± 0.04	48.36 ± 7.33	0.89 ± 0.23	12.72 ± 3.01
Ad2 infection	6.48 ± 0.90	0.85 ± 0.10	43.89 ± 8.46	1.01 ± 0.28	10.76 ± 2.51
HFD	8.36 ± 1.37*	2.06 ± 0.63*	57.26 ± 9.31*	0.56 ± 0.13*	21.81 ± 3.31*
Ad36 infection	5.71 ± 1.52	0.70 ± 0.01*	48.33 ± 10.06	1.23 ± 0.33*	12.56 ± 2.13

Note**:** *HFD* high fat diet, *HOMA-IR* Homeostasis model assessment of insulin resistance. * *P* < 0.05, compared with the Control group

The results of IPGTT showed that the glucose concentration increased rapidly after intraperitoneal injection of glucose in each group, and gradually decreased after reaching the peak at 15 min. In the HFD group, glucose concentration and area under the curve (AUC) at 0,15, 30, 60 and 120 min after injection were significantly higher than those of the Control group (*P* < 0.05). There was no significant difference in blood glucose or AUC between the Ad36-infection group and the Control group (*P* > 0.05, Fig. 1b and c), suggesting that the rats in the Ad36 infection group had better glucose tolerance than the HFD group.

ITT results showed that the concentrations of glucose and AUC in the HFD group at 20, 40, 90 and 120 min after injection were higher than those in the Control group (*P* < 0.05). However, there was no significant difference in blood glucose level and AUC between the Ad36 infection group and the Control group (*P* > 0.05, Fig. 1d and e) at each time point after glucose injection, suggesting that Ad36-infected rats were obese but the insulin sensitivity was not compromised.

### Ad36 infection does not cause liver steatosis and adipocyte hypertrophy

**Fig. 2:**
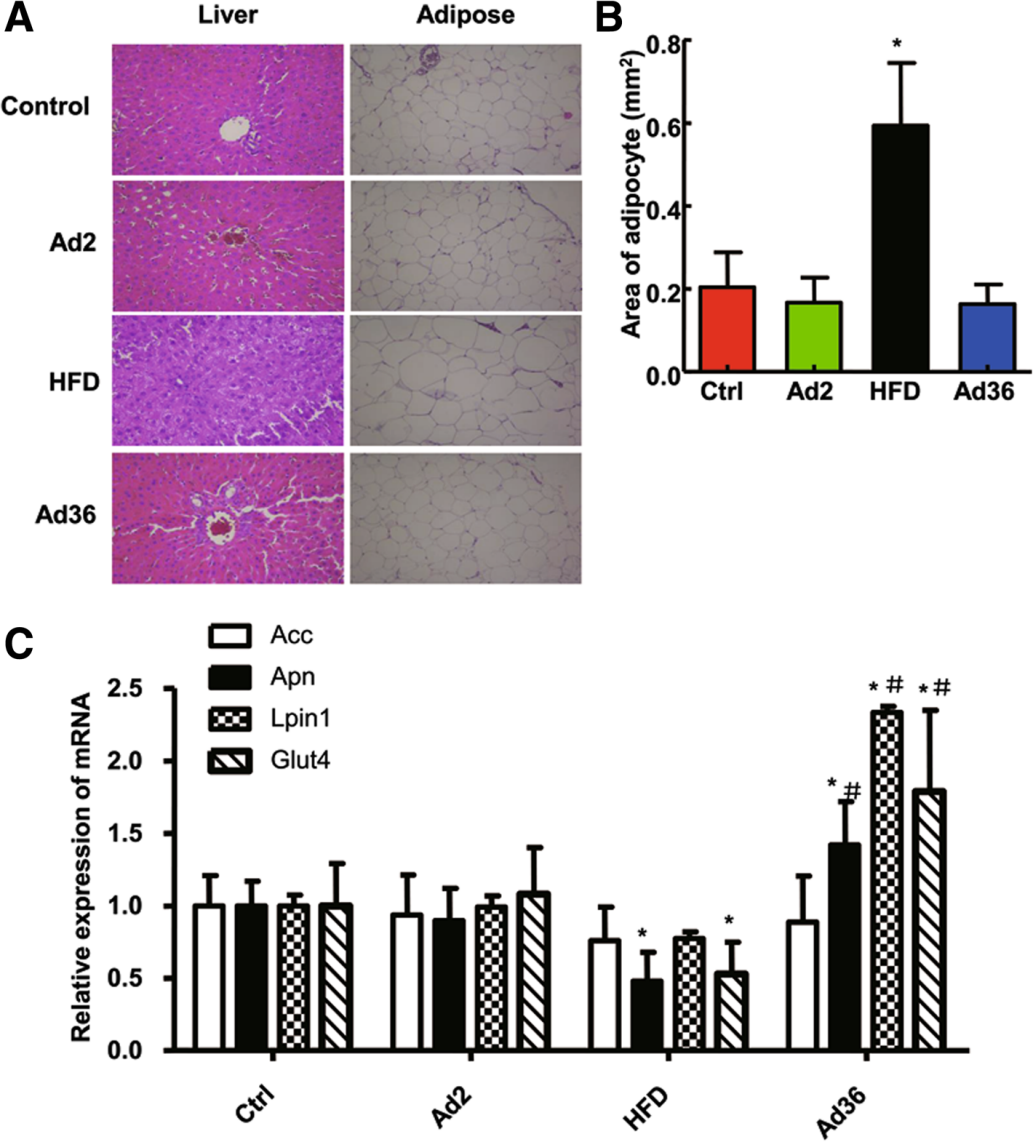
The effect of Ad36 infection on the lipid deposition in liver and the related gene expressions. **a** After infected with Ad36, the liver and adipose tissues were collected and sectioned. The H&E staining was performed and the sections were photographed. **b** The area of adipocytes after Ad36 infection was calculated from the HE stained sections. **c** The expression levels of Acc, Adipoq, Lpin1 and Glut4 in the adipose tissues after Ad36 infection were determined by quantitative PCR. HFD was used as the positive control, and Ad2 was used as the negative control. * *P* < 0.05, compared with the control group; ^#^ *P* < 0.05, compared with the HFD group

To determine whether Ad36 induced obesity may cause lipid heterotopic deposition, HE staining was performed. Liver lobule structure was clear and complete in the liver tissue sections in the blank control group, Ad2 infection group, and Ad36 infection group. The hepatocyte structure was complete, the cytoplasm was uniformly red stained, and no fat droplets, denaturation and necrosis were observed. In the HFD group, there was a large number of small lipid droplets in the hepatocytes were at the 20th week. A small number of hepatocytes showed balloon-like changes but inflammatory cell infiltration and focal necrosis were not observed (Fig. 2a). Compared with the blank control group, the adipocyte area in the adipose tissue of the HFD group was significantly increased (*P* < 0.05), while the Ad36-induced obese rats had smaller adipocyte area than the blank control group, but the difference was not statistically significant (*P* > 0.05, Fig. 2b). The above results indicate that although the body weight of rats increases after Ad36 infection, there is no fat deposition in liver cells.

### Changes of gene expression in the adipose tissue of Ad36-induced obese rats

To detect the difference of adipocytokines expression between Ad36 and HFD induced obese rats, qPCR was performed. Compared with the control group, the expression of Glut4, Lpin1 and Adipoq in the adipose tissue of Ad36 group increased significantly, while the expression of Glut4 and Adipoq in the adipose tissue of HFD group decreased (*P* < 0.05). Compared with the HFD group, the expression of Glut4, Lpin1 and Adipoq in the adipose tissue of Ad36-induced obese rats was also significantly increased (*P* < 0.05, Fig. 2c).

Taken together, these results indicated that although the body weight of rats increased after Ad36 infection, the blood glucose and blood triglyceride levels did not increase, insulin sensitivity was normal, and lipid ectopic deposition did not occur, which were unlike high-fat induced obesity. This may be due to changes in the level of adipocytokines that affect the metabolism of glucose and lipids.

### Ad36-induced hADSC shows increased transcriptional activity of PPARγ and downstream target genes

**Fig. 3:**
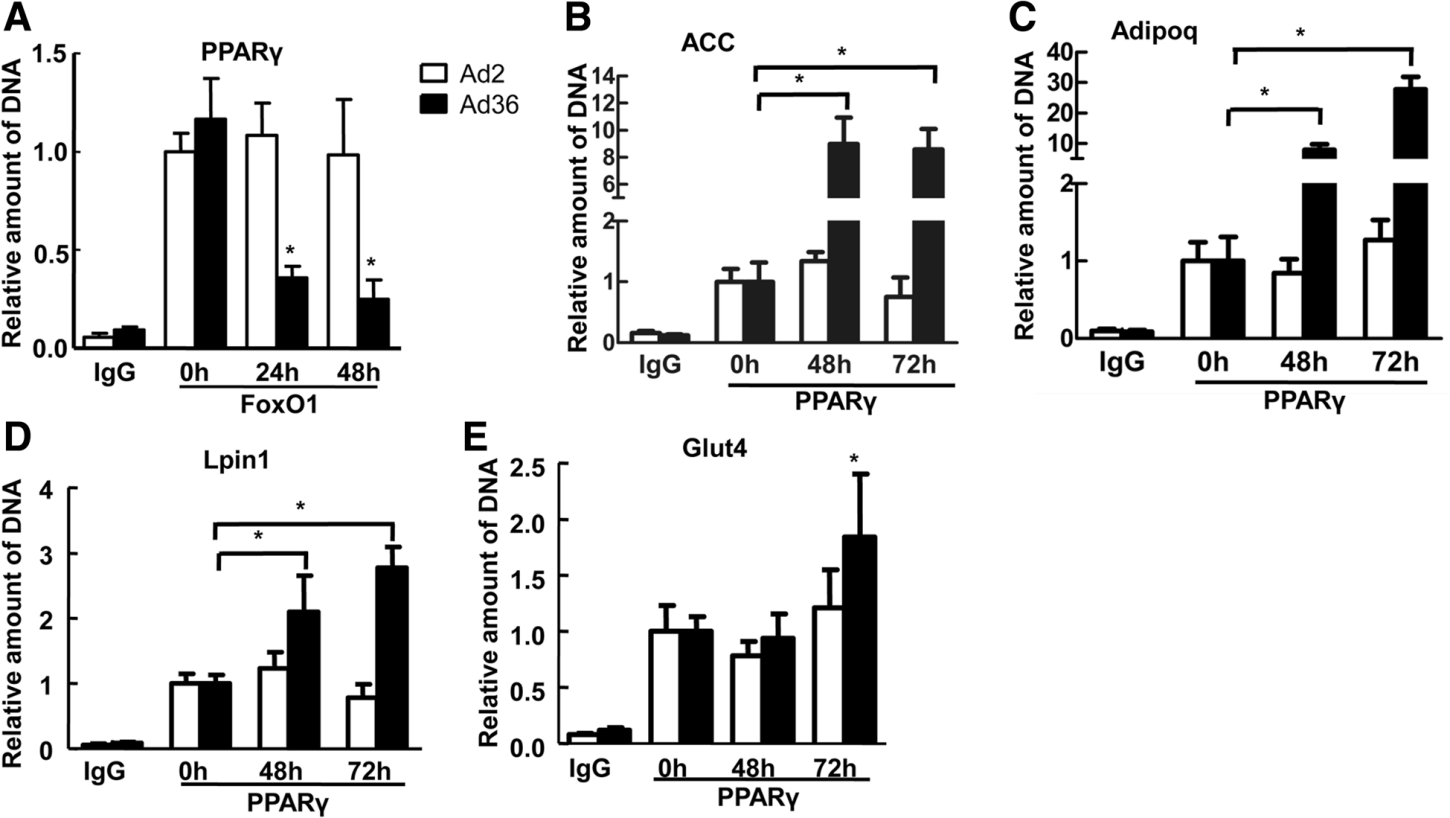
Ad36-induced hADSC showed increased PPARγ and downstream target gene transcription activity. **a** Ad36 reduced FoxO1 binding to PPARγ promoter region. **b**-**e** Ad36 increased PPARγ binding ability to Acc, Adipoq, Lpin1 and Glut4 promoter regions. Ad2 was used as the negative control. * *P* < 0.05, compared with the 0-h non-infection group

To determine the effect of Ad36 on transcriptional activity of adipocytokines in vitro, ChIP was performed. In the in vitro experiment of Ad36-induced hADSC adipogenesis, the ChIP -PCR product was calibrated using the amount of input and the results showed that PPARγ promoter region DNA fragments were amplified from the precipitated chromatin fragments by FoxO1 antibody (Fig. 3a). Compared with the 0-h non-infection group, the amount of the precipitated PPARγ decreased by 3.27 times after 24 h of induction with Ad36, and the immunoprecipitation amount decreased by 4.7 times after 48 h of induction (Fig. 3a). After precipitation with anti-PPARγ antibody, the quantitative analysis showed that compared with the 0-h non-infection group, the amount of precipitated Acc (Fig. 3b), Adipoq (Fig. 3c) and Lpin1 (Fig. 3d) after 48 h of Ad36 induction increased by 8.99, 7.86, and 2.10 folds, respectively. The amount of precipitated Acc (Fig. 3b), Adipoq (Fig. 3c), Lpin1 (Fig. 3d) and Glut4 (Fig. 3e) after 72 h of Ad36 induction increased by 8.58, 27.77, 2.78 and 1.84 folds, respectively, and the differences were statistically significant (*P* < 0.05). The results showed that the binding rate between FoxO1 and PPARγ promoter region was significantly decreased by Ad36, but the binding rate of PPARγ to Acc, Adipoq, Lpin1 and Glut4 was enhanced.

### Effect of Ad36 induction on the expression of FoxO1 and PPARγ genes

**Fig. 4:**
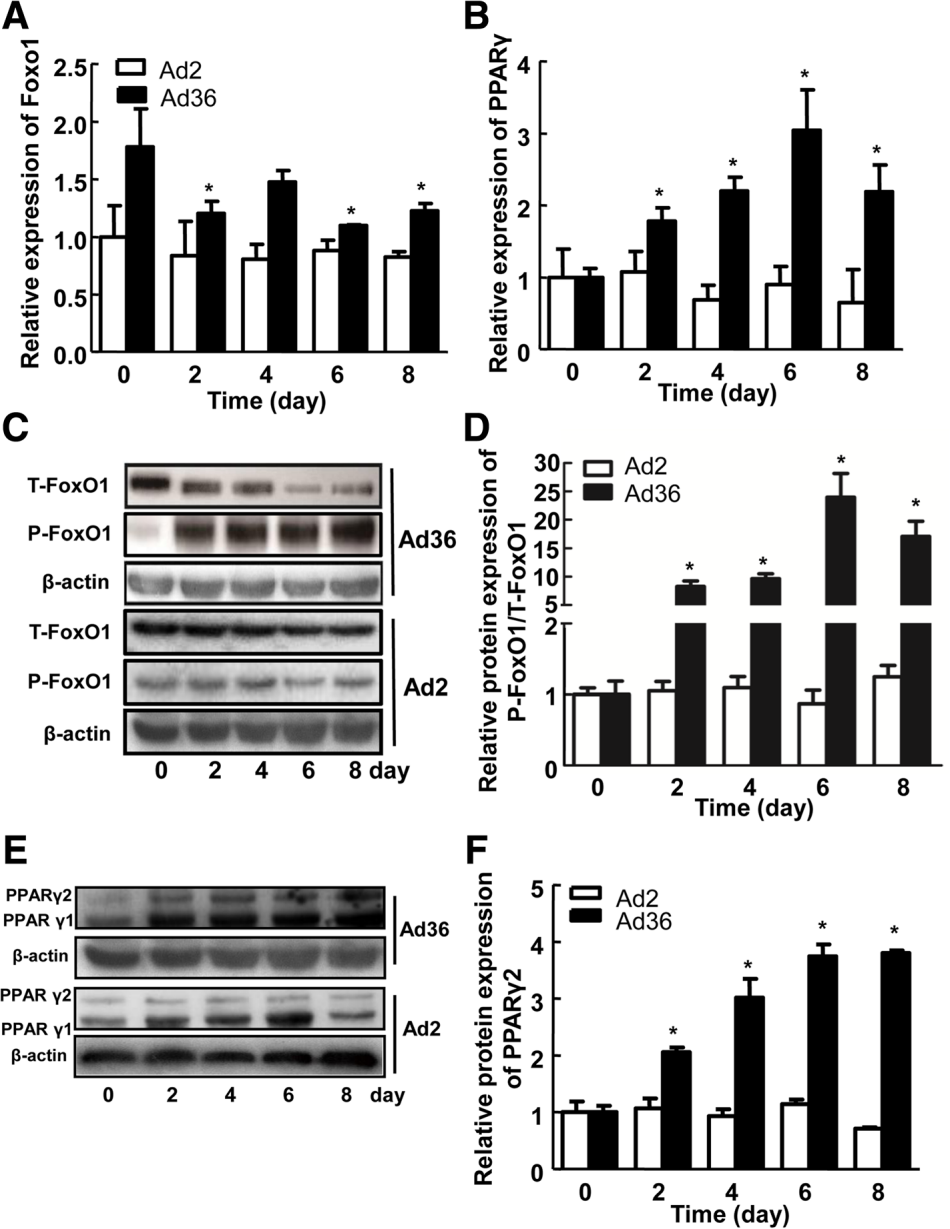
The effect of Ad36 induction on FoxO1 and PPARγ gene expression. **a**-**b** The effect of Ad36 induction on T-FoxO1 and PPARγ gene expression; (**c**-**d**) Changes of T-FoxO1 and P-FoxO1 protein expression after Ad36 induction; (**e**-**f**) Changes of PPARγ protein expression after Ad36 induction. Ad2 was used as the negative control. * *P* < 0.05, compared with the 0-day non-infection group

The hADSCs were infected with 5MOI of Ad36 and Ad2, and the expression of FoxO1 and PPARγ mRNA was detected on the 0th, 2nd, 4th, 6th and 8th days by qPCR. Compared with that on the 0th day, the FoxO1 mRNA expression levels on the 2nd, 6th, and 8th days after Ad36 infection decreased, and the difference was statistically significant (*P* < 0.05) (Fig. 4a). The mRNA expression levels of PPARγ on the 2nd, 4th, 6th and 8th day were 1.78, 2.20, 3.04, and 2.19 times of that on the 0th day, and the difference was statistically significant (*P* < 0.05, Fig. 4b).

Protein expression changes of FoxO1 and PPARγ were detected by Western blot. The amount of FoxO1 also decreased, but the phosphorylated FoxO1 protein increased on the 2nd, 4th, 6th and 8th days, compared to the 0th day (Fig. 4c and d), and thus the ratio of p-FoxO1/FoxO1 significantly increased (*P* < 0.05). The PPARγ gene has two transcripts, i.e. PPARγ1 and PPARγ2, of which PPARγ2 is specifically expressed in adipocytes and is often used as a marker gene for the differentiation of adipocytes [18]. The results of this experiment showed that the expression of PPARγ2 was increased on the 2nd, 4th, 6th and 8th day, compared with that on the 0th day (*P* < 0.05) (Fig. 4e and f). These results suggest that Ad36 increases the expression of P-FoxO1 and PPARγ2, which affect adipose cell differentiation.

### Ad36 induces adipogenesis of hADSC by changing the expression of glycolipid metabolism genes

**Fig. 5:**
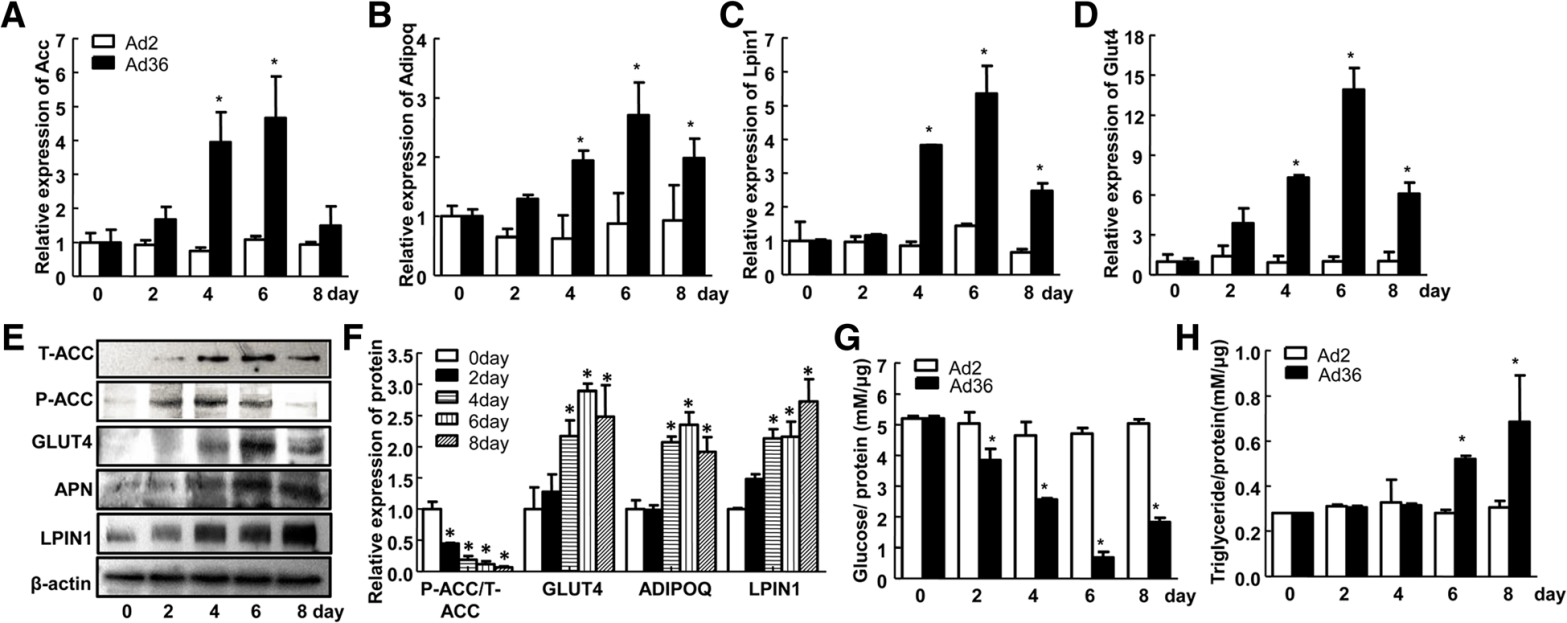
Ad36 induces adipogenesis of hADSC by increasing the expression of glucolipid metabolism associated genes. **a**-**d** Changes of Acc, Adipoq, Lpin1 and Glut4 mRNA expression after Ad36 induction; (**e**) Changes in the expression level of the downstream target proteins of PPARγ after Ad36 induction; (**f**) Quantification of the Western blot bands of the downstream target proteins of PPARγ; (**g**) Changes in the concentration of glucose in the medium; (**h**) Changes in intracellular triglyceride levels. Ad2 was used as the negative control.* *P* < 0.05, compared with the 0-day non-infection group

To further detect the expression of adipocytokines, qPCR was performed. Compared with that on Day 0, the expression of Acc gene was significantly increased on Day 4 and Day 6 after Ad36 infection (Fig. 5a) (*P* < 0.05). The mRNA levels of Adipoq, Lpin1 and Glut4 were all increased on the 4th, 6th and 8th day after Ad36 infection (*P* < 0.05, Fig. 5b, c and d). Additionally, Western blot was also used. Compared with that on Day 0, the protein expression levels of LPIN1 and ADIPOQ were significantly increased on Day 4, 6 and 8 after Ad36 induction (*P* < 0.05), and the ratio of phosphorylated ACC to total ACC gradually decreased on Day 2, 4, 6 and 8 (*P* < 0.05). The expression of GLUT4 protein in cell membrane was significantly increased on Day 4, 6 and 8 after Ad36 infection (*P* < 0.05, Fig. 5e and f).

The content of glucose in the culture supernatant of hADSC after Ad36 infection was detected and was corrected by the total protein content in the culture dish, which indirectly reflected the glucose uptake ability of the cells. Compared with that on day 0, the glucose concentration in the supernatant of the Ad36-infected hADSC on the 2nd, 4th, 6th and 8th days were reduced (*P* < 0.05), and the glucose concentration on the 6th day reached the lowest (Fig. 5g). The intracellular triglyceride content was determined by the GPO-PAP method and was corrected for total cellular protein. The content of intracellular triglyceride was significantly increased on the 6th and 8th day after the induction of Ad36 (*P* < 0.05), and the content of triglyceride on the 8th day was the highest (Fig. 5h). These results suggest that Ad36 induces adipogenesis of hADSC by effecting the expression of glycolipid metabolism genes.

### Wortmannin inhibits adipogenesis of hADSC

**Fig. 6:**
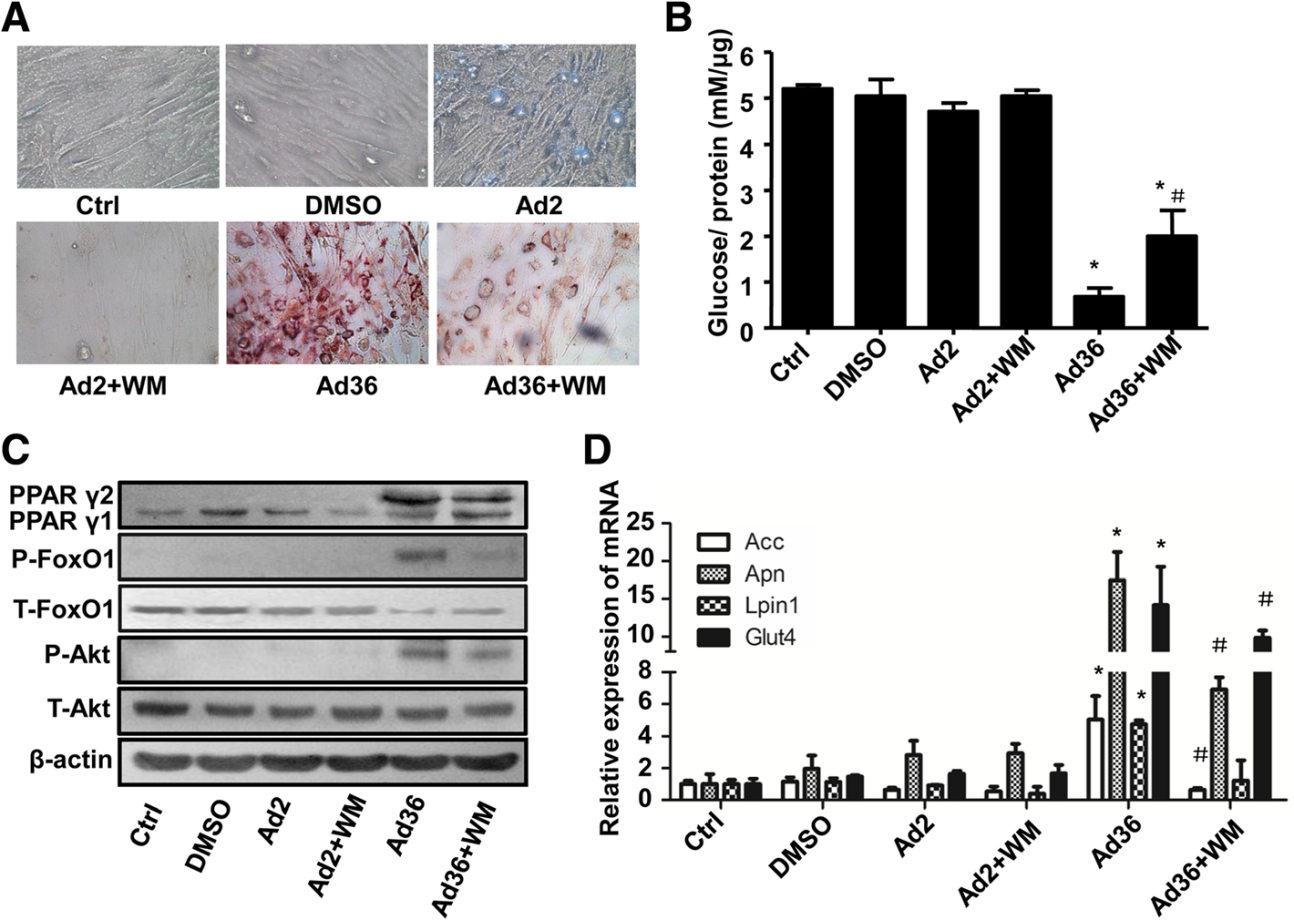
Wortmannin inhibits adipogenesis of hADSC. **a** Oil red O staining was performed to detect the lipid droplet in hADSC; (**b**) The concentration of glucose in the culture medium; (**c**) The protein expression levels of Akt, FoxO1 and PPARγ were detected by Western blot; (**d**) The mRNA expression levels of Acc, Adipoq, Lpin1 and Glut4 were measured by quantitative PCR. Ad2 was used as the negative control. * *P* < 0.05, compared with the control group; ^#^ *P* < 0.05, compared with the Ad36 infection group

The results of the oil red O staining on the 6th day after the intervention showed that the lipid droplets in the Ad36 + Wortmannin group were obviously reduced compared to those in the Ad36 infection group (Fig. 6a). The level of glucose in the culture medium was significantly decreased after the induction of Ad36 (*P* < 0.05). After adding Wortmannin (Ad36 + Wortmannin group), the glucose content in the medium was higher than that in the Ad36 infection group (*P* < 0.05, Fig. 6b). These suggest that Wortmannin inhibits the uptake of glucose in Ad36-induced cells.

Western blot was used to detect the effect of Wortmannin on PI3K-induced target protein Akt. The results showed that the expression of P-Akt protein, P-FoxO1 protein and PPARγ2 in the Ad36 + Wortmannin group was lower than that in the Ad36 infection group (Fig. 6c). The mRNA expression levels of the downstream target genes of PPARγ, including Acc, Adipoq, Lpin1 and Glut4, significantly decreased after Wortmannin intervention compared with the Ad36 infection group (*P* < 0.05, Fig. 6d). These results suggest that Wortmannin may inhibit Ad36-induced adipogenesis through the PI3K/Akt signaling pathway.

## Discussion

Obesity is considered to be a non-infectious, chronic, low-grade inflammatory state [19]. Although obesity is an important cause of insulin resistance, not all obese individuals have insulin resistance. Viral infection is considered to be a cause of obesity, which breaks the traditional understanding of the causes of obesity. The results of our previous study found that the Ad36 infection rate in Uygur obese population was significantly higher than that in non-obese populations, but blood triglyceride levels were reduced after Ad36 infection [20].

This study used Wistar rats as the research objects to establish two obesity models respectively induced by HFD and Ad36 infection. The body weight, clinical biochemical indicators, pathological morphology and gene expression were analyzed to evaluate the differences between the two types of obesity models. The results showed that the body weight of the HFD and Ad36-infected group was significantly increased, which is consistent with the previous findings of Dhurandhar et al. [21]. Obesity caused by a HFD shows excessive accumulation of triglycerides in visceral fat cells and increased cell tension [22]. In order to maintain its own steady-state regulatory role, the body tends to redistribute fat and/or increase catabolism, resulting in a large amount of free fatty acid (FFA) overflow and increase of blood FFA levels [23]. The increase in FFA may cause dyslipidemia on the one hand, and on the other hand it may inhibit the use of glucose, reduce the sensitivity of insulin, and promote insulin resistance [24]. In addition, adipose hypertrophy causes excessive feedback on fat storage signals, resulting in disorders of adipocytokines and decrease in the ability of surrounding tissues to take up and use glucose [25]. The excessive glucose continuously stimulates islet β cells to secrete large amounts of insulin, which can induce the synthesis of triglycerides from fatty acids and glucose and lead to the accumulation of triglycerides in the liver [26]. Therefore, there is fat ectopic metastasis and hepatic steatosis in HFD rats [27]. However, levels of blood glucose and triglycerides did not increase significantly in obesity model induced by Ad36 infection. In addition, IPGTT and ITT results did not indicate impaired glucose tolerance and insulin sensitivity. HE staining results showed that Ad36-induced obese rats did not exhibit excessive lipid accumulation in fat cells and ectopic deposition of lipids in the liver.

FoxO1 and PPARγ are important transcription factors that affect the differentiation of adipocyte. FoxO1 expression and protein function are regulated at multiple levels, including regulation of transcription, post-translational modification and protein-protein interaction. Post-translational modifications such as phosphorylation, ubiquitination and acetylation directly affect the subcellular localization, transcriptional activity and stability of FoxO1 protein. Under basic conditions, FoxO1 inhibits the expression of PPARγ by binding to the promoter of PPARγ, thereby inhibiting the differentiation of adipocytes [28]. Ad36-induced Akt activation significantly increases the phosphorylation of FoxO1 protein [12]. Phosphorylated FoxO1 protein is translocated from the nuclear to the cytoplasm, thus weakening the transcriptional inhibition of PPARγ [29]. In this study, there was no significant change in PPARγ1 protein expression after Ad36 infection, but PPARγ2 protein was significantly higher, suggesting PPARγ2 is the main transcription factor promoting the adipocyte differentiation [18].

As predicted by the online software (http://alggen.lsi.upc.es/cgi-bin/promo_v3/promo/promoinit.cgi?dirDB=TF_8.3) (data not shown), PPARγ may bind to the promoter regions of Acc, Adipoq, Lpin1 and Glut4 genes. Our results by ChIP assay found that the binding and transcriptional activities of PPARγ to Acc, Adipoq and Lpin1 promoter DNA were significantly enhanced after the induction by Ad36. Maeda et al. [30] showed that adiponectin knockout rats developed significant symptoms of insulin resistance, such as hyperglycemia and hyperinsulinemia, after feeding high-sugar and HFD. This insulin resistance was significantly improved after supplementation with adiponectin. In this study, obesity induced by HFD rats showed abnormal clinical manifestations such as hyperglycemia, hypertriglyceridemia, impaired glucose tolerance and impaired insulin resistance. The expression of Adipoq in serum and adipose tissue was significantly lower than that in the control group. However, the expression of adiponectin in the serum and adipose tissue of Ad36-induced obese rats was increased.

Lpin, including Lpin1, Lpin2 and Lpin3, is a recently discovered gene family that bi-directionally regulates body fat metabolism [31]. High expression of Lpin1 in adipose tissue and skeletal muscle can cause obesity [32], but the insulin sensitivity is different in the two tissues. Lipin 1 mRNA levels in abdominal visceral adipose tissue were negatively correlated with body mass index, body fat ratio, plasma triglyceride, and plasma leptin levels, and positively correlated with PPARγ and adiponectin mRNA levels in abdominal visceral and subcutaneous adipose tissue [33]. Our results showed that the expression of Lpin1 in adipose tissue was significantly increased after Ad36 infection. This may be due to that Lpin1 promotes the accumulation of triglyceride in adipose tissue and preventing the lipid ectopic deposition in skeletal muscle or islet β cells; Or because Lpin1 promotes the expression of Glut4, so that fat cells can take glucose more efficiently [34].

The above results indicate that Ad36 infection changes the expression levels of the transcription factors of FoxO1 and PPARγ, and a series of genes related to glucose and lipid metabolism, promotes Ad36-induced differentiation of hADSC into adipocytes, and increases glucose uptake capacity. It is reported that Ad36 E4orf1 binds to Dlg-1 protein through its PBM to form a complex and penetrates the cell membrane to activate the PI3K/Akt signaling pathway [10, 11, 35]. To confirm whether Ad36 affects FoxO1 and downstream gene expression through activated protein kinase Akt, we used the PI3K inhibitor WM. The results of oil red O staining on the 6th day of WM intervention showed that Ad36 induced rounding of the cells, and 70 to 80% of the cells showed red lipid droplets and started fusion. Compared with the Ad36 alone group, the phosphorylated Akt protein decreased after WM treatment, which reduced the phosphorylation level of FoxO1 protein. FoxO1 in the nucleus inhibited the expression of PPARγ, and the lipid droplets in the cells were significantly reduced. WM intervention reduced the adipogenic ability of hADSC induced by Ad36.

## Conclusions

Ad36 increases the expression of Acc, Lpin1, Glut4 and Adipoq possibly by activating the PI3K/Akt/FoxO1/PPARγ signal pathway, promoting the synthesis of fatty acids and triglycerides, and improving insulin sensitivity.

## Abbreviations

Ad36: Adenovirus type 36
AUC: area under the curve
Dlg-1: Drosophila Disc Large-1
hADSCs: human adipose derived stem cells
HFD: high fat diet
IPGTT: intraperitoneal glucose tolerance test
ITT: insulin tolerance test
PBM: PDZ domain binding motif
T2DM: type 2 diabetes mellitus
WM: Wortmannin

## References

[1] Atkinson RL, Dhurandhar NV, Allison DB, Bowen RL, Israel BA, Albu JB, Augustus AS (2005). Human adenovirus-36 is associated with increased body weight and paradoxical reduction of serum lipids. Int J Obes.

[2] Dhurandhar NV (2011). A framework for identification of infections that contribute to human obesity. Lancet Infect Dis.

[3] Dhurandhar NV (2013). Insulin sparing action of adenovirus 36 and its E4orf1 protein. J Diabetes Complicat.

[4] Hainer V, Zamrazilova H, Kunesova M, Bendlova B, Aldhoon-Hainerova I (2015). Obesity and infection: reciprocal causality. Physiol Res.

[5] Villavicencio F, Valladares M (2017). The potential contribution of adenovirus 36 to the development of obesity. Rev Med Chil.

[6] Sapunar J, Fonseca L, Molina V, Ortiz E, Barra MI, Reimer C, Charles M, Schneider C, Ortiz M, Brito R, et al: Adenovirus 36 seropositivity is related to obesity risk, glycemic control, and leptin levels in Chilean subjects. 2019. 10.1038/s41366-019-0321-4 30659258

[7] Hegde V, Na HN, Dubuisson O, Burke SJ, Collier JJ, Burk D, Mendoza T, Dhurandhar NV (2016). An adenovirus-derived protein: a novel candidate for anti-diabetic drug development. Biochimie.

[8] Kocazeybek B, Dinc HO, Ergin S, Saribas S, Ozcabi BT, Cizmecigil U, Altan E, Atalik K, Yuksel P, Taner Z (2017). Evaluation of Adenovirus-36 (Ad-36) antibody seropositivity and adipokine levels in obese children. Microb Pathog.

[9] Vangipuram SD, Yu M, Tian J, Stanhope KL, Pasarica M, Havel PJ, Heydari AR, Dhurandhar NV (2007). Adipogenic human adenovirus-36 reduces leptin expression and secretion and increases glucose uptake by fat cells. Int J Obes.

[10] Rogers PM, Fusinski KA, Rathod MA, Loiler SA, Pasarica M, Shaw MK, Kilroy G, Sutton GM, McAllister EJ, Mashtalir N (2008). Human adenovirus Ad-36 induces adipogenesis via its E4 orf-1 gene. Int J Obes.

[11] Dhurandhar EJ, Dubuisson O, Mashtalir N, Krishnapuram R, Hegde V, Dhurandhar NV (2011). E4orf1: a novel ligand that improves glucose disposal in cell culture. PLoS One.

[12] Kong K, Kumar M, Taruishi M, Javier RT (2014). The human adenovirus E4-ORF1 protein subverts discs large 1 to mediate membrane recruitment and dysregulation of phosphatidylinositol 3-kinase. PLoS Pathog.

[13] Andjelković M, Alessi DR, Meier R, Fernandez A, Lamb NJ, Frech M, Cron P, Cohen P, Lucocq JM, Hemmings BA (1997). Role of translocation in the activation and function of protein kinase B. J Biol Chem.

[14] Nagai S, Matsumoto C, Shibano M, Fujimori K (2018). Suppression of fatty acid and triglyceride synthesis by the flavonoid Orientin through decrease of C/EBPδ expression and inhibition of PI3K/Akt-FOXO1 signaling in adipocytes. Nutrients.

[15] Armoni M, Harel C, Karni S, Chen H, Bar-Yoseph F, Ver MR, Quon MJ, Karnieli E (2006). FOXO1 represses peroxisome proliferator-activated receptor-gamma1 and -gamma2 gene promoters in primary adipocytes. A novel paradigm to increase insulin sensitivity. J Biol Chem.

[16] Jiao Y, Aisa Y, Liang X, Nuermaimaiti N, Xian G, Zhang Z, Guan Y (2016). Regulation of PPARγ and CIDEC expression by adenovirus 36 in adipocyte differentiation. Mol Cell Biochem.

[17] Li M, Tang Y, Wu L, Mo F, Wang X, Li H, Qi R, Zhang H, Srivastava A, Ling C: The hepatocyte-specific HNF4alpha/miR-122 pathway contributes to iron overload-mediated hepatic inflammation. 2017, 130:1041–1051. 10.1182/blood-2016-12-755967 PMC5570677 28655781

[18] Tontonoz P, Hu E, Spiegelman BM (1994). Stimulation of adipogenesis in fibroblasts by PPAR gamma 2, a lipid-activated transcription factor. Cell.

[19] Gregor MF, Hotamisligil GS (2011). Inflammatory mechanisms in obesity. Annu Rev Immunol.

[20] Ponterio E, Gnessi L (2015). Adenovirus 36 and obesity: an overview. Viruses.

[21] Dhurandhar NV, Israel BA, Kolesar JM, Mayhew GF, Cook ME, Atkinson RL (2000). Increased adiposity in animals due to a human virus. Int J Obes Relat Metab Disord.

[22] Bays H (2014). Central obesity as a clinical marker of adiposopathy; increased visceral adiposity as a surrogate marker for global fat dysfunction. Curr Opin Endocrinol Diabetes Obes.

[23] Ali AH, Koutsari C, Mundi M, Stegall MD, Heimbach JK, Taler SJ, Nygren J, Thorell A, Bogachus LD, Turcotte LP (2011). Free fatty acid storage in human visceral and subcutaneous adipose tissue: role of adipocyte proteins. Diabetes.

[24] Morigny P, Houssier M, Mouisel E, Langin D (2016). Adipocyte lipolysis and insulin resistance. Biochimie.

[25] Yazici D, Sezer H (2017). Insulin resistance, obesity and lipotoxicity. Adv Exp Med Biol.

[26] Tchkonia T, Thomou T, Zhu Y, Karagiannides I, Pothoulakis C, Jensen MD, Kirkland JL (2013). Mechanisms and metabolic implications of regional differences among fat depots. Cell Metab.

[27] Gaborit B, Dutour A (2016). Ectopic fat deposition and diabetes mellitus. J Am Coll Cardiol.

[28] Zou P, Liu L, Zheng L, Liu L, Stoneman RE, Cho A, Emery A, Gilbert ER, Cheng Z (2014). Targeting FoxO1 with AS1842856 suppresses adipogenesis. Cell Cycle.

[29] Van Der Heide LP, Hoekman MF, Smidt MP (2004). The ins and outs of FoxO shuttling: mechanisms of FoxO translocation and transcriptional regulation. Biochem J.

[30] Maeda N, Shimomura I, Kishida K, Nishizawa H, Matsuda M, Nagaretani H, Furuyama N, Kondo H, Takahashi M, Arita Y (2002). Diet-induced insulin resistance in mice lacking adiponectin/ACRP30. Nat Med.

[31] Kim J, Lee YJ, Kim JM, Lee SY, Bae MA, Ahn JH, Han DC, Kwon BM (2016). PPARgamma agonists induce adipocyte differentiation by modulating the expression of Lipin-1, which acts as a PPARgamma phosphatase. Int J Biochem Cell Biol.

[32] Csaki LS, Dwyer JR, Li X, Nguyen MH, Dewald J, Brindley DN, Lusis AJ, Yoshinaga Y, de Jong P, Fong L (2014). Lipin-1 and lipin-3 together determine adiposity in vivo. Mol Metab.

[33] Chang YC, Chang LY, Chang TJ, Jiang YD, Lee KC, Kuo SS, Lee WJ, Chuang LM (2010). The associations of LPIN1 gene expression in adipose tissue with metabolic phenotypes in the Chinese population. Obesity (Silver Spring).

[34] van Harmelen V, Ryden M, Sjolin E, Hoffstedt J (2007). A role of lipin in human obesity and insulin resistance: relation to adipocyte glucose transport and GLUT4 expression. J Lipid Res.

[35] Dhurandhar EJ, Krishnapuram R, Hegde V, Dubuisson O, Tao R, Dong XC, Ye J, Dhurandhar NV (2012). E4orf1 improves lipid and glucose metabolism in hepatocytes: a template to improve steatosis & hyperglycemia. PLoS One.

